# Characterization of *Salmonella* Gallinarum isolates from backyard poultry by polymerase chain reaction detection of invasion (*inv*A) and *Salmonella* plasmid virulence (*spv*C) genes

**DOI:** 10.14202/vetworld.2017.814-817

**Published:** 2017-07-23

**Authors:** Susmita Pal, Samir Dey, Kunal Batabyal, Abhiroop Banerjee, Siddhartha Narayan Joardar, Indranil Samanta, Devi Prasad Isore

**Affiliations:** Department of Veterinary Microbiology, West Bengal University of Animal and Fishery Sciences, Belgachia, Kolkata - 700 037, West Bengal, India

**Keywords:** *inv*A, polymerase chain reaction, *Salmonella* Gallinarum, *Salmonella* plasmid virulence *C*, virulence genes

## Abstract

**Aim:**

The aim was to characterize *Salmonella enterica* serovar Gallinarum isolated from backyard poultry by polymerase chain reaction (PCR) detection of virulence genes invasion (*inv*A) and *Salmonella* plasmid virulence C (*spv*C).

**Materials and Methods:**

Two strains of *Salmonella* serovar Gallinarum isolates used in this study were obtained from an outbreak of fowl typhoid in backyard Vanaraja fowl. PCR technique was used for detection of *inv*A and *spv*C genes using standard methodology. The *inv*A PCR product from one representative isolate was sequenced and compared with other related *Salmonella* serovars in GenBank data.

**Results:**

*Salmonella* Gallinarum produced expected amplicons of *inv*A and *spv*C gene products. Nucleotide sequence of 285 bp *inv*A gene was deposited in GenBank with accession no. KX788214. Sequence analysis of *inv*A gene was found conserved in *Salmonella* serovars and demonstrated 100% homology with closely related serovars of *Salmonella*.

**Conclusion:**

Invasion gene (*inv*A) was found to be highly conserved in *Salmonella* Gallinarum and highly similar with closely related serovars. The isolates also contained plasmid-mediated spvC gene indicating possession of virulence plasmid.

## Introduction

Fowl typhoid (FT) is disease of major economic significance in many countries of Asia, Africa, Central and South America [1]. It is an endemic disease of poultry in India with occasional outbreaks [2-4]. The pathogen *Salmonella enterica* serovar Gallinarum can colonize and cause disease in various domestic and wild birds. The pathogen can get transmitted by both horizontal and vertical routes. The majority of virulence genes of *Salmonella* are clustered in a region distributed over the chromosome, called *Salmonella* pathogenicity islands (SPI). A total of 19 SPI have been described with SPI-1 to SP-5 being present in most serovars and others were being less widely distributed [5]. Besides, one large plasmid of approximately 85 kb in *Salmonella* Gallinarum have the ability of strains to produce high mortality in chickens [6] and *Salmonella* plasmid virulence (*spv*) locus that carries the *spv* genes were reported to be present in *Salmonella* Gallinarum-Pullorum and few other non-typhoid *Salmonella* serovars, namely, *Salmonella* Abortusovis, *Salmonella* Choleraesuis, *Salmonella* Dublin, *Salmonella* Enteritidis, and *Salmonella* Typhimurium, and *Salmonella* Sendai [7] and absent in typhoid serovars Typhi and Paratyphi [8].

The chromosomally located invasion gene (*inv*A) being thought to trigger the invasion of *Salmonellae* into cultured epithelial cells [9], while an operon (*spv*RABCD) in plasmid containing five genes, involved in intra-macrophage survival of *Salmonella* [10]. Characterization of *Salmonella* serovars has been carried out previously by various researchers by polymerase chain reaction (PCR) assay of different virulence factors [11-13], but the study was less reported with *Salmonella* Gallinarum particularly from backyard poultry.

The current study was aimed to characterize *Salmonella* Gallinarum obtained from backyard poultry by detection of virulence genes *inv*A and *spv*C.

## Materials and Methods

### Ethical approval

As per the Committee for the Purpose of Control and Supervision on Experiments on Animals (CPCSEA) guidelines, this study does not require ethical approval from Institute Animal Ethics Committee.

### Bacterial strains

Two isolates of *Salmonella* Gallinarum (WBSG-1, WBSG-2) obtained from the Department of Veterinary Microbiology, West Bengal University of Animal and Fishery Sciences, Kolkata, from an outbreak of FT in Vanaraja fowl were used. The isolates were serotyped with antigenic structure (9,12:-:-) at National *Salmonella* and *Escherichia* Centre, Kasauli, India.

### Preparation of culture lysate

Bacterial culture lysate was prepared as described previously [14] with little modification. 1 ml of overnight broth culture of *Salmonella* Gallinarum was taken in a sterile 1.5 ml microcentrifuge tube (Tarsons, India) and centrifuged at 6000 rpm for 5 min. The pellet was washed twice with Tris-ethylenediaminetetraacetic acid (EDTA) buffer and was re-suspended in 1 ml Tris-EDTA buffer. Then, the culture was boiled for 10 min followed by chilling in ice. The cell debris was removed by centrifugation at 6000 rpm for 5 min. Then, the supernatant was stored at −20°C for further use as template DNA.

### PCR assay

*Salmonella* specific primers described previously [15], the forward primer S139 and reverse primer S141 (Table-1) based on the *invA* gene of *Salmonella* were used. The amplification of the *inv*A gene fragment was carried out as described earlier with little modifications [16]. The PCR was carried out with a 25 µl amplification mixture consisting of 3 µl template DNA, 5 µl of ×5 GoTaq^®^ Flexi buffer, 0.5 µl of deoxynucleotide triphosphates (10 mM each), 1.6 µl of 25 mM MgCl_2_, 1 µl of 10 µM each primer and 0.3 µl of GoTaq^®^ DNA polymerase (Promega, USA), and 12.6 µl nuclease free water. Amplification was conducted in a thermocycler (Mastercycler personal, Eppendorf, Germany). The cycle condition consisted an initial denaturation 94°C for 1 min followed by 35 cycles of denaturation at 94°C for 60 s, annealing at 64°C for 30 s, and elongation at 72°C for 30 s with 7 min final extension period at 72°C. The amplified products were visualized by agarose gel electrophoresis containing 1.5% w/v agarose (SRL, India) with ethidium bromide (0.5 µg/ml) and detected by gel documentation system (UVP, UK).

**Table-1:** Oligonucleotides (primers) used for detection of virulence genes (*invA* and s*pvC*) of *Salmonella* Gallinarum.

Genes	Primer	Oligonucleotides (5’-3’)	Amplification product (bp)	References
*invA*	S139	GTG AAA TTA TCG CCA CGT TCG GGC AA	284	[15]
	S141	TCATCGCACCGTCAAAGGAACC		
*spv*C	SPV-1	ACTCCTTGCACAACCAAATGCGGA	571	[7]
	SPV-2	TGTCTTCTGCATTTCGCCACCATCA		

*spv*=*Salmonella plasmid* virulence

For *spv*C gene, another set primer was used (Table-1) [7]. The amplification conditions for the *spv*C gene fragment being similar as described for *inv*A gene except the annealing temperature was 58°C.

### Nucleotide sequencing of invA gene

Positive amplification from a PCR reaction of *inv*A gene from one representative isolate (WBSG1) was purified with DNA gel/PCR purification mini kit (Xcelris, India). Both strands of purified PCR product were sequenced with forward and reverse primers for *inv*A gene in an ABI 3730 XL automated sequencer (Applied Biosystems) in custom sequencing facility of Xcelris, India. Sequence obtained was analyzed, and homology searches were conducted using the BLAST algorithm (www.ncbi.nlm.nih.gov/BLAST).

## Results

**Figure-1:**
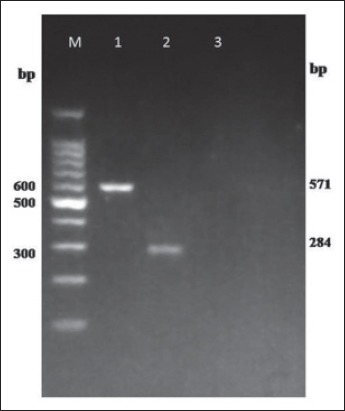
Polymerase chain reaction amplification of virulence genes *invA* and *Salmonella* plasmid virulence C (*spv*C) of *Salmonella* Gallinarum isolates. Lane M: 100 bp DNA ladder, Lane 1: *inv*A gene (strain WBSG1), Lane 2: *spvC* gene (strain WBSG2), Lane 3: Negative control.

Amplicons of *inv*A and spvC virulence genes were observed in agarose gel as ~284 bp and ~571 bp products, respectively (Figure-1).

Nucleotide sequence of *inv*A gene of *Salmonella* Gallinarum strainWBSG1 obtained in this study was analyzed and 285 bp sequences deposited with NCBI under GenBank accession number KX788214. Sequence alignment with BLAST revealed that *inv*A gene of *Salmonella* Gallinarum strain WBSG1 was highly similar (100%) with some other poultry serovars such as *Salmonella enterica* serovar Gallinarum strain 9184 (accession no. CP019035.1) and *Salmonella* Enteritidis strain OLF 00D 98987-1 (accession no. CP011942.1) isolated elsewhere.

## Discussion

Detection of invasion gene of *Salmonella* by PCR-based assays may be useful for rapid pathogen identification as well. Molecular identification of *Salmonella* sp. with *inv*A gene primer set S139-S141 conforms to be international standard [17-19] with very high specificity [15]. However, choosing suitable primers are important as the primer sets targeting different sequences within *inv*A gene [7], often resulted in non-specific amplification with the fecal and gut-associated bacteria [20]. In one study, *Salmonella* isolates belonging to serotypes Anatum, Enteritidis and Amsterdam were also reported negative for the *inv*A gene using those primers [21].

High prevalence of *inv*A virulence gene in *Salmonella* serovars has also been reported by other workers [22,23]. We found *inv*A gene was 100% similar with other *Salmonella* serovars. Other studies also reported similar results [24], which were expected since the invasion gene (*inv*A) is conserved among *Salmonella* serovars. Serovar Enteritidis, Dublin, and Gallinarum were reported to be closely related where serovar Dublin and Gallinarum diverging independently from an Enteritidis-like ancestor [25].

In this study, both *Salmonella* Gallinarum isolates were positive to *spv*C gene. This finding was similar with a study in Kashmir where all isolates of *Salmonella* from poultry harbored virulence genes *inv*A and *spv*C [26]. However, less prevalence of *spv* genes was noticed in *Salmonella* serovars by several workers [9,27,28]. In a study with 37 *Salmonella* comprising serovar Enteritidis (*n*=12) and Typhimurium (*n*=24) originated from pork and slaughterhouse environment, all have produced 284 bp *inv*A gene, but no *spv*C gene [23]. In another study, a high prevalence (88.6%) of *spv*A, *spv*B, and *spv*C genes was observed in *S. Enteritidis* from poultry source [13]. One main function of the *spv* operon is to potentiate the systemic spread of the pathogen [29], and these genes can restore pathogenicity for systemic spread in plasmid-cured strains [30]. The *spv* region contains three genes required for the virulence phenotype in mice; the positive transcriptional regulator *spv*R and two structural genes *spv*B and *spv*C [8]. Mutations in *spv*C and *spv*D genes cause various (allele-specific) defects in *Salmonella* virulence [31].

## Conclusion

Invasion gene (*inv*A) was found to be highly conserved in *Salmonella* Gallinarum and highly similar with closely related serovars. The isolates also contained *spv*C gene indicating possession of plasmid virulence.

## Authors’ Contributions

SP, KB, and SD planned and designed the study. The experiment was conducted by SP, AB, and IS, data analysis was performed by SNJ, SD, and DPI. All authors participated in the draft and revision of the manuscript. All authors read and approved the final manuscript.
